# Supplementary material for: RORα controls hepatic lipid homeostasis via negative regulation of PPARγ transcriptional network
Source: Nat Commun. 2017 Jul 31;8:162. doi: 10.1038/s41467-017-00215-1 (PMC5534431; doi:10.1038/s41467-017-00215-1)
Supplement: Supplementary file 1 — Supplementary Information [file 41467_2017_215_MOESM1_ESM.pdf]

File name: Supplementary Information
Description: Supplementary figures.

File name: Supplementary Data 1
Description: related to Figure 3a. Summary of the alignment results for mRNA-sequencing data. Among the raw reads generated from Illumina Hiseq-2500, the reads after trimming low quality and adapter sequences were used for the alignment. On average, each 98.5 % of the reads (Mapped reads and Mapping rate) were aligned to a reference mouse genome (GRCm38). Of the mapped reads, 92.1 % (Unique mapping rate) of the mapped reads were aligned to unique location (Uniquely mapped reads) in the genome. Exom Coverage denotes the fold of coverage of the mapped reads for the annotated exon region.

File name: Supplementary Data 2
Description: related to Figure 3a. List of genes affected by depletion of RORα. Up- and down-regulation of the genes affected by ROR⍰ depletion in the following three comparisons are shown together with their $\log_2$-fold-changes and P-values: 1) CD-fed RORα^{LKO} versus CD-fed ROR$\alpha^{f/f}$ (KO/WT_{CD}), 2) HFD-fed RORα^{LKO} versus HFD-fed ROR$\alpha^{f/f}$ (KO/WT_{HFD}), and 3) their $\log_2$-fold-change differences ((KO/WT_{HFD})/(KO/WT_{CD})). For each gene, its group number (Group) derived from the three comparisons is also included.

File name: Supplementary Data 3
Description: related to Figure 3b. GOBPs and KEGG pathways represented by the genes in Groups 1-4. The GOBPs and KEGG pathways represented by the genes in Groups 1-8 are shown. For each GOBP or KEGG pathway term, the count of the genes involved in the term and the enrichment P-values for the term are shown. The GOBPs and KEGG pathways with P-value<0.05 and Count >2 were selected as representative ones.

File name: Supplementary Data 4
Description: related to Figure 3c. Key transcription factors significantly regulating the genes in Group 1. TFs significantly enriched by the genes in Group 1 (P-value < 0.01) are shown. For each TF, the number of genes and the list of genes targeted by the TF are also provided.

File name: Supplementary Data 5
Description: Primer list for qRT-PCR and ChIP analysis.

File name: Peer review file
Description:

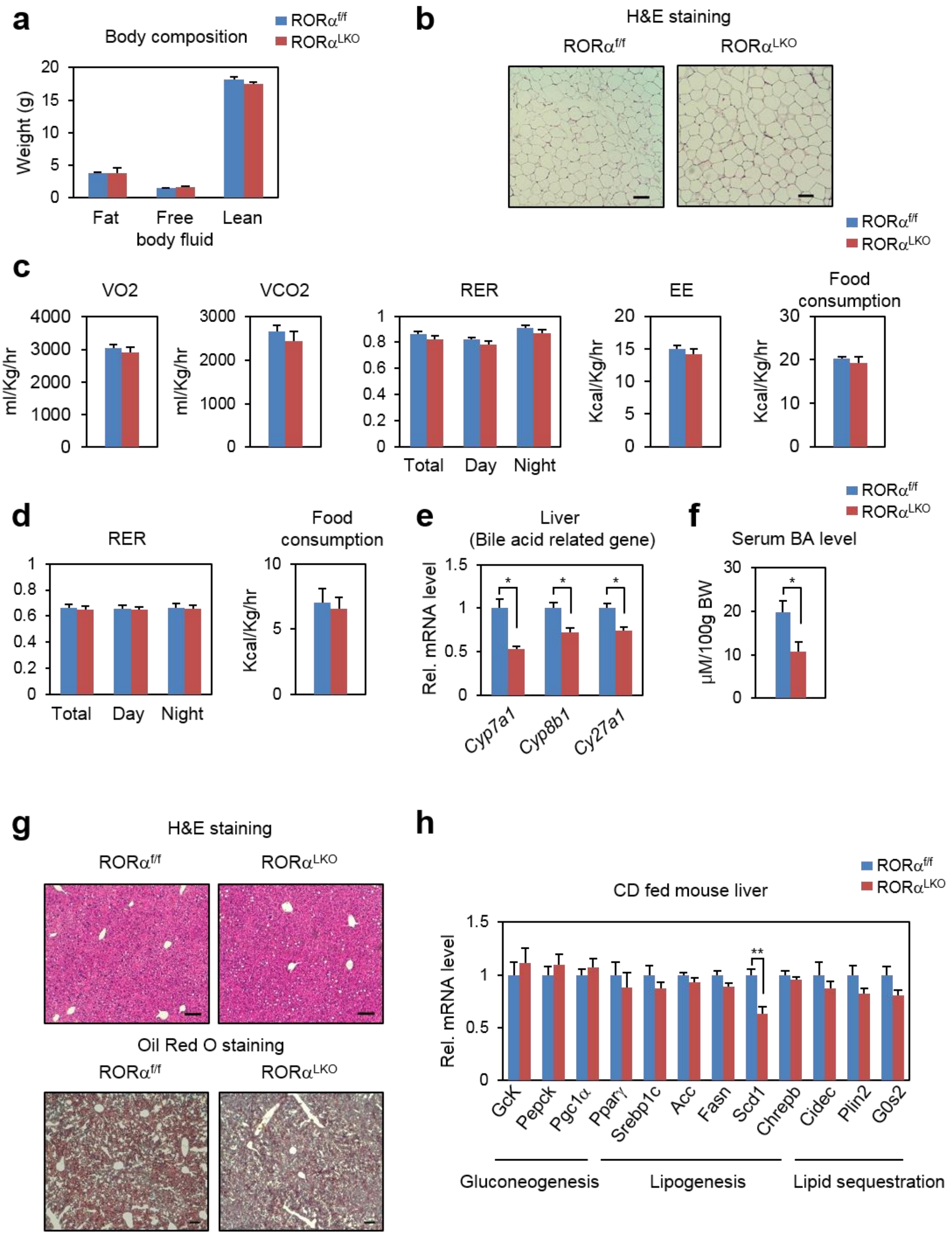
a
Body composition
RORα f/f
RORα LKO
Weight (g)
20
15
10
5
0
Fat
Free body fluid
Lean
b
H&E staining
RORα f/f
RORα LKO
c
VO2
ml/Kg/hr
4000
3000
2000
1000
0
VCO2
ml/Kg/hr
3000
2000
1000
0
RER
1
0.8
0.6
0.4
0.2
0
Total
Day
Night
EE
Kcal/Kg/hr
20
15
10
5
0
Food consumption
Kcal/Kg/hr
30
20
10
0
d
RER
Total
Day
Night
Food consumption
Kcal/Kg/hr
10
5
0
e
Liver (Bile acid related gene)
Rel. mRNA level
1.5
1
0.5
0
Cyp7a1
Cyp8b1
Cy27a1
f
Serum BA level
μM/100g BW
30
20
10
0
g
H&E staining
RORα f/f
RORα LKO
Oil Red O staining
RORα f/f
RORα LKO
h
CD fed mouse liver
Rel. mRNA level
1.5
1
0.5
0
Gck
Pepck
Pgc1α
Pparγ
Srebp1c
Acc
Fasn
Scd1
Chrepb
Cidec
Plin2
G0s2
Gluconeogenesis
Lipogenesis
Lipid sequestration

Supplementary Figure 1. Characterization of RORα$^{f/f}$ and RORα^{LKO}mice fed CD and HFD

(**a**) Body composition analysis of RORα$^{f/f}$ and RORα^{LKO}mice fed CD for 10 weeks (n=6/group). (**b**) Representative image of eWAT from RORα$^{f/f}$ and RORα^{LKO} mice fed CD for 10 weeks stained with hematoxylin and eosin. Scale bar, 100 μm. (**c**) Metabolic cage studies were performed in RORα$^{f/f}$ and RORα^{LKO} mice fed CD for 10 weeks (n=6 mice/group). O2 consumption (VO2), CO2 production (VCO2), respiratory exchange ratio (RER), energy expenditure (EE) and food consumption were represented (left to right, in order). (**d**) Metabolic cage studies were performed in RORα$^{f/f}$ and RORα^{LKO} mice fed HFD for 10 weeks (n=5-6 mice/group). Respiratory exchange ratio (RER) and food consumption were represented (left to right, in order). (**e**) Expression levels of bile acid related genes in liver extract from RORα$^{f/f}$ and RORα^{LKO} mice fed HFD for 10 weeks (n=7/group) as determined by qRT-PCR. Expression was normalized to 36B4 expression. (**f**) Bile acid level of serums that were collected from RORα$^{f/f}$ and RORα^{LKO} mice fed HFD for 10 weeks (n=6/group). BW, body weight. Data expressed as mean ± SEM. Statistical analysis was performed using Student's unpaired t-test. *p<0.05. (**g**) Representative liver histological section images of RORα$^{f/f}$ and RORα^{LKO} mice fed CD for 10 weeks stained with hematoxylin and eosin (upper panel) and Oil Red O (bottom panel). Scale bar, 100 μm. (**h**) Hepatic gene expression profile involved in metabolism from the livers of RORα$^{f/f}$ and RORα^{LKO} mice (n=5/group) fed CD for 10 weeks as determined by qRT-PCR. Expression was normalized to 36B4 expression. Data expressed as mean ± SEM. Statistical analysis was performed using Student's unpaired t-test. **p<0.01.

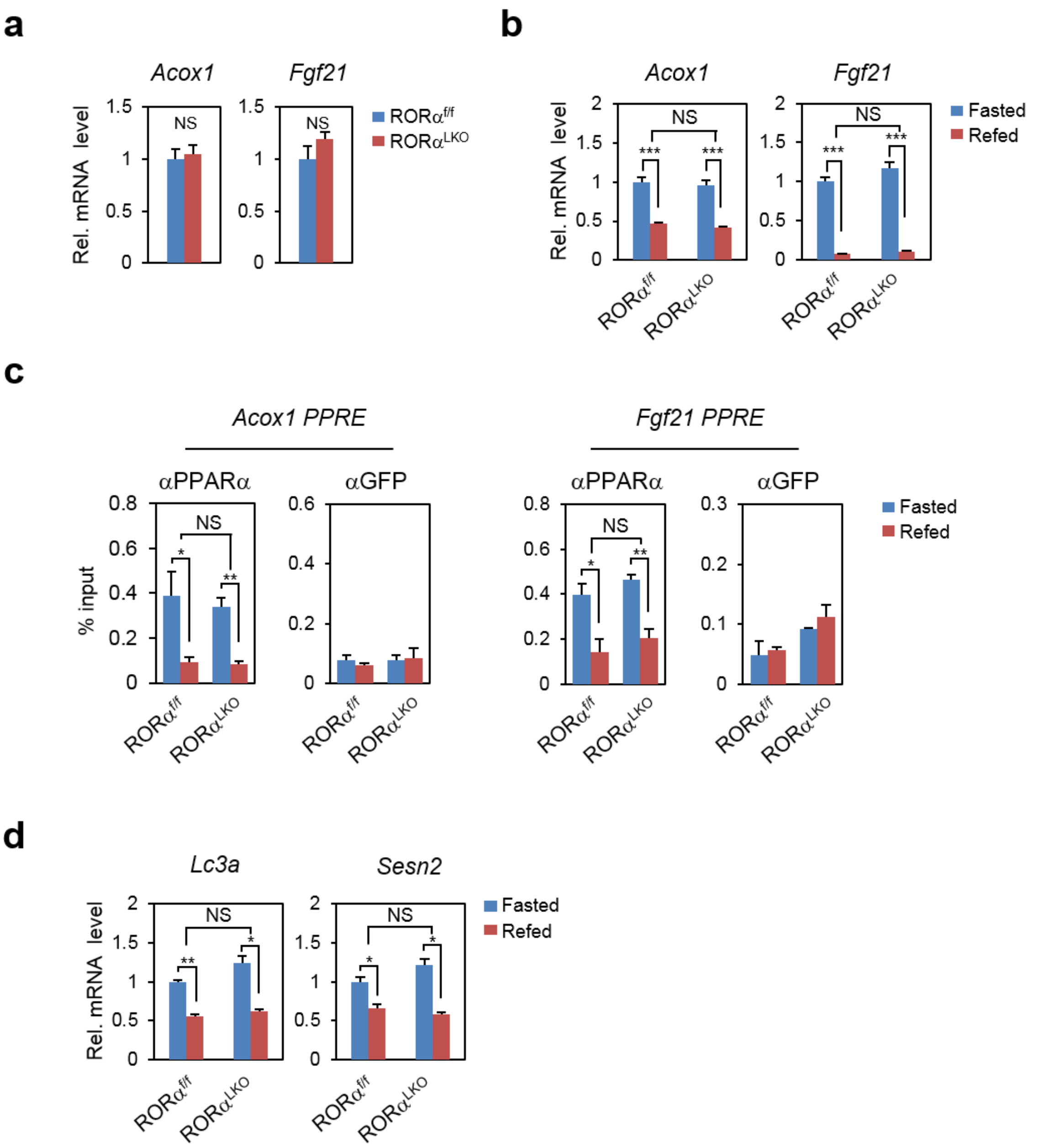

Supplementary Figure 2. PPARα and RORα do not affect their roles in the mice liver with each other

(**a**) Expression levels of PPARα target genes in liver from RORα$^{f/f}$ and RORα^{LKO} mice fed CD or HFD for 10 weeks (n=7-9/group) as determined by qRT-PCR. Expression was normalized to 36B4 expression. Data expressed as mean ± SEM. Statistical analysis was

performed using Student's unpaired t-test. NS=Non-Significant. (**b-d**) 24 h fasted and 24 h refed after 24 h fasting ROR$\alpha^{f/f}$ and RORα^{LKO} mice were euthanized to collect livers. (**b**) Expression levels of PPARα target genes in liver from ROR$\alpha^{f/f}$ and RORα^{LKO} mice fasted or refed (n=3/group) as determined by qRT-PCR. Expression was normalized to 36B4 expression. (**c**) ChIP assays were performed on the *Acox1* and *Fgf21* promoters in liver extract form ROR$\alpha^{f/f}$ and RORα^{LKO} mice fasted or refed (n=3/group). Promoter occupancy by PPARα and GFP was analyzed. (**d**) Expression levels of autophagy-related PPARα target genes in liver from ROR$\alpha^{f/f}$ and RORα^{LKO} mice fasted or refed (n=3/group) as determined by qRT-PCR. Expression was normalized to 36B4 expression. Data expressed as mean ± SEM. Statistical analysis was performed using two-way ANOVA. *p<0.05, **p<0.01, ***p<0.001, NS=Non-Significant.

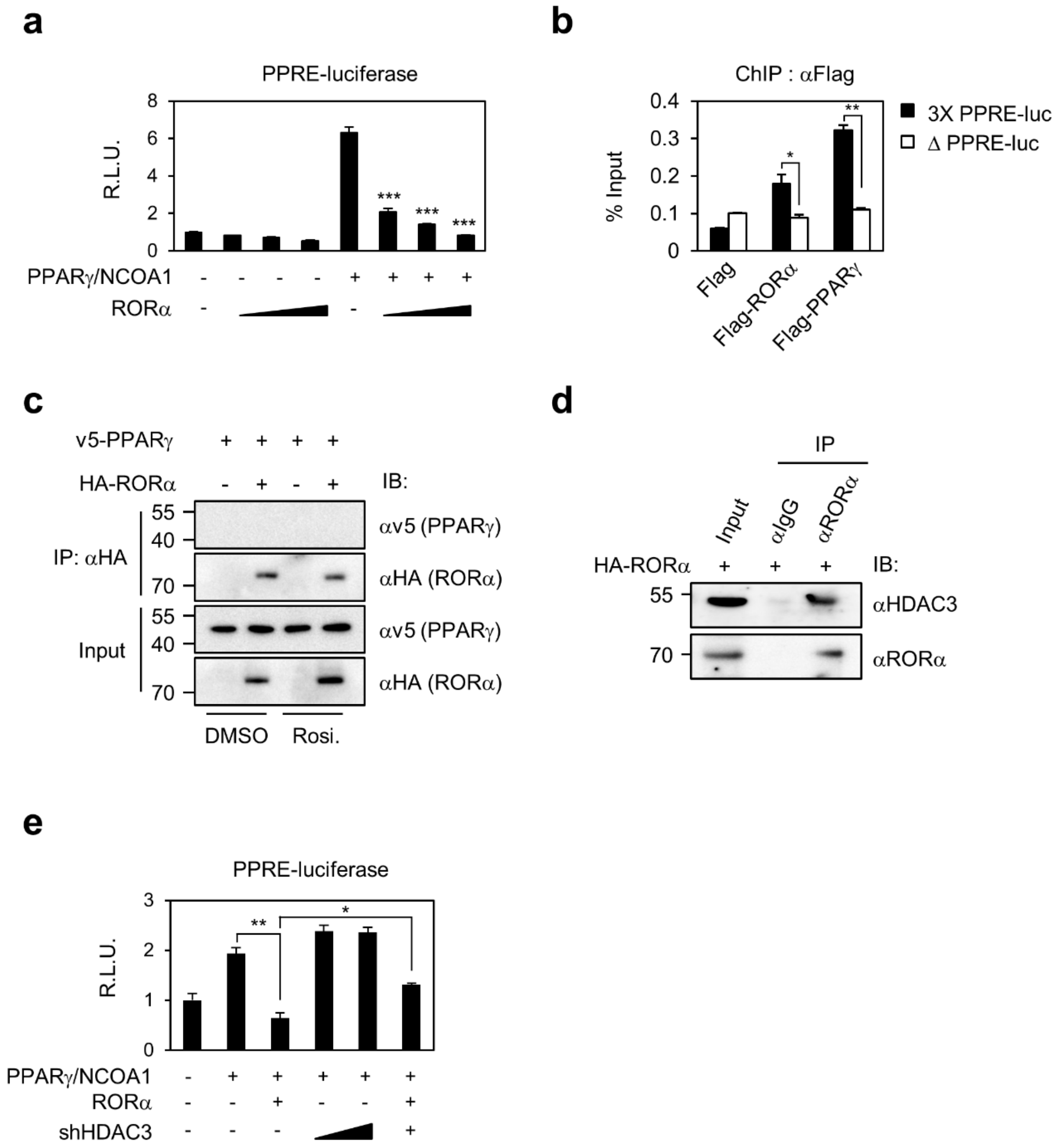

Supplementary Figure 3. RORα interacts with HDAC3 to repress PPARγ transcriptional activity with p160 family coactivator.

(**a**) Effect of overexpression of RORα on PPRE-luciferase reporter activity with coactivator NCOA1. Data expressed as mean ± SEM. Statistical analysis was performed using one-way ANOVA followed by Tukey's post hoc analysis. ***$p<0.001$, compared to PPARγ/PGC1α group. (**b**) WT PPRE promoter and PPRE deleted mutant promoter containing luciferase

reporter plasmid and Flag/Flag-RORα/Flag-PPARγ plasmid were transfected into Hep3B cells. ChIP assays were performed on promoter region of reporter plasmid in Hep3B cells. Promoter occupancy by Flag was analyzed. Data expressed as mean ± SEM. Statistical analysis was performed using Student's unpaired t-test. *$p<0.05$, **$p<0.01$. (**c**) Co-immunoprecipitation assay was performed to detect the interaction between RORα and PPARγ of HEK293T cells. (**d**) Co-immunoprecipitation assay was performed to detect the interaction between RORα and HDAC3 of HEK293T cells. (**e**) Effect of knockdown of HDAC3 with coactivator NCOA1 on PPRE-luciferase reporter activity. Data expressed as mean ± SEM. Statistical analysis was performed using one-way ANOVA followed by Tukey's post hoc analysis. *$p<0.05$, **$p<0.01$.

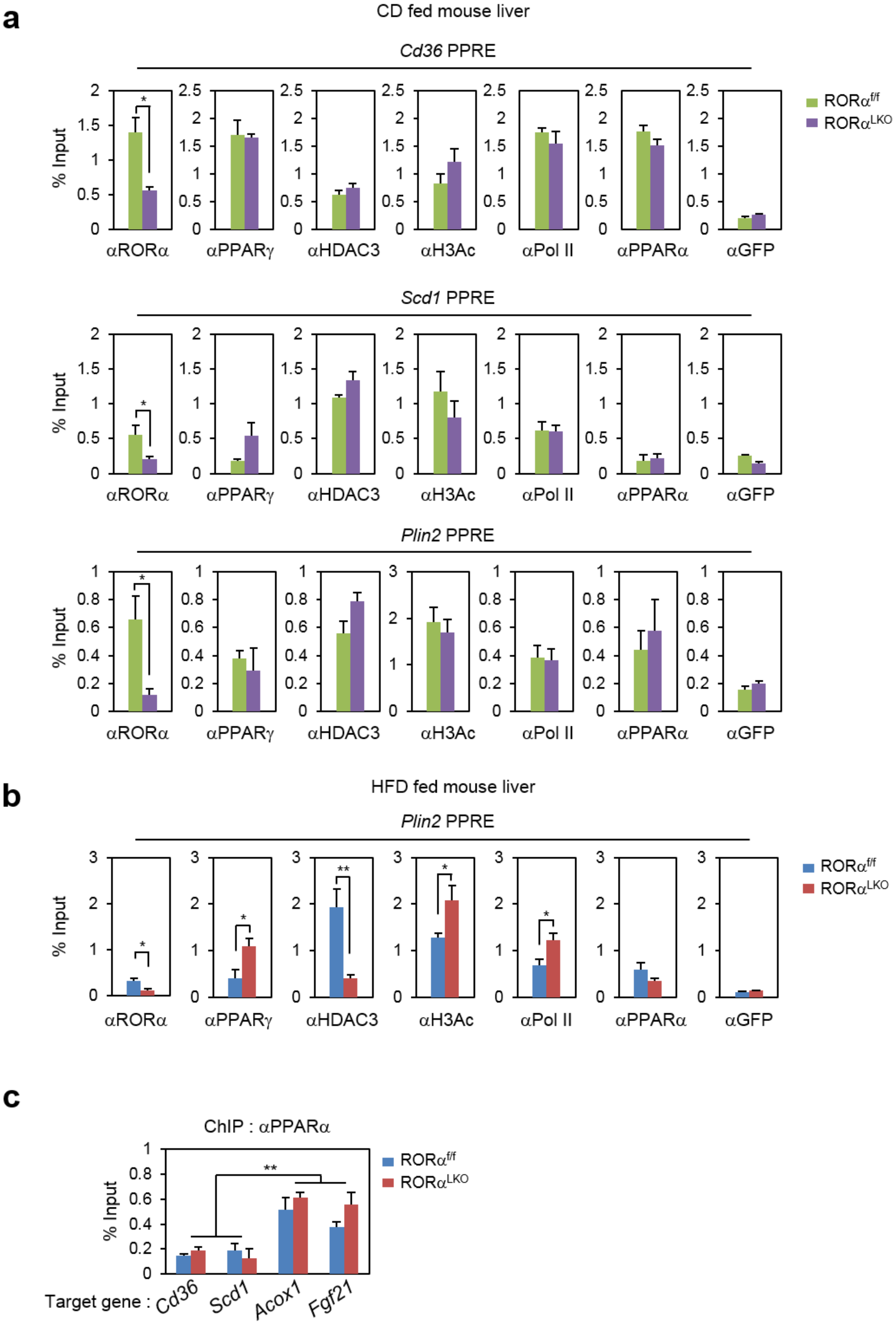
a
CD fed mouse liver
Cd36 PPRE
Scd1 PPRE
Plin2 PPRE
% Input
αRORα
αPPARγ
αHDAC3
αH3Ac
αPol II
αPPARα
αGFP
RORα f/f
RORα LKO
b
HFD fed mouse liver
Plin2 PPRE
c
ChIP : αPPARα
Target gene :
Cd36
Scd1
Acox1
Fgf21

Supplementary Figure 4. The recruitment of PPARγ to the PPARγ target gene promoters is similar between RORα$^{f/f}$ and RORα^{LKO} mice fed CD unlike mice fed HFD.

(**a**) ChIP assays were performed on the *Cd36*, *Scd1* and *Plin2* promoters in liver extract form RORα$^{f/f}$ and RORα^{LKO} mice fed CD for 10 weeks (n=3/group). Promoter occupancy by RORα, PPARγ, PGC1α, HDAC3, H3Ac, Pol II, PPARα and GFP was analyzed. (**b**) ChIP assays were performed on the *Plin2* promoters in liver extract form RORα$^{f/f}$ and RORα^{LKO} mice fed HFD for 10 weeks (n=3/group). Promoter occupancy by RORα, PPARγ, PGC1α, HDAC3, H3Ac, Pol II, PPARα and GFP was analyzed. Data expressed as mean ± SEM. Statistical analysis was performed using Student's unpaired t-test. *$p<0.05$, **$p<0.01$.

(**c**) ChIP assays were performed on the *Cd36, Scd1, Acox1 and Fgf21* promoters in liver extract form RORα$^{f/f}$ and RORα^{LKO} mice fed HFD for 10 weeks (n=3/group). Promoter occupancy by PPARα was analyzed. Data expressed as mean ± SEM. Statistical analysis was performed using two-way ANOVA followed by Tukey's post hoc analysis. **$p<0.01$.

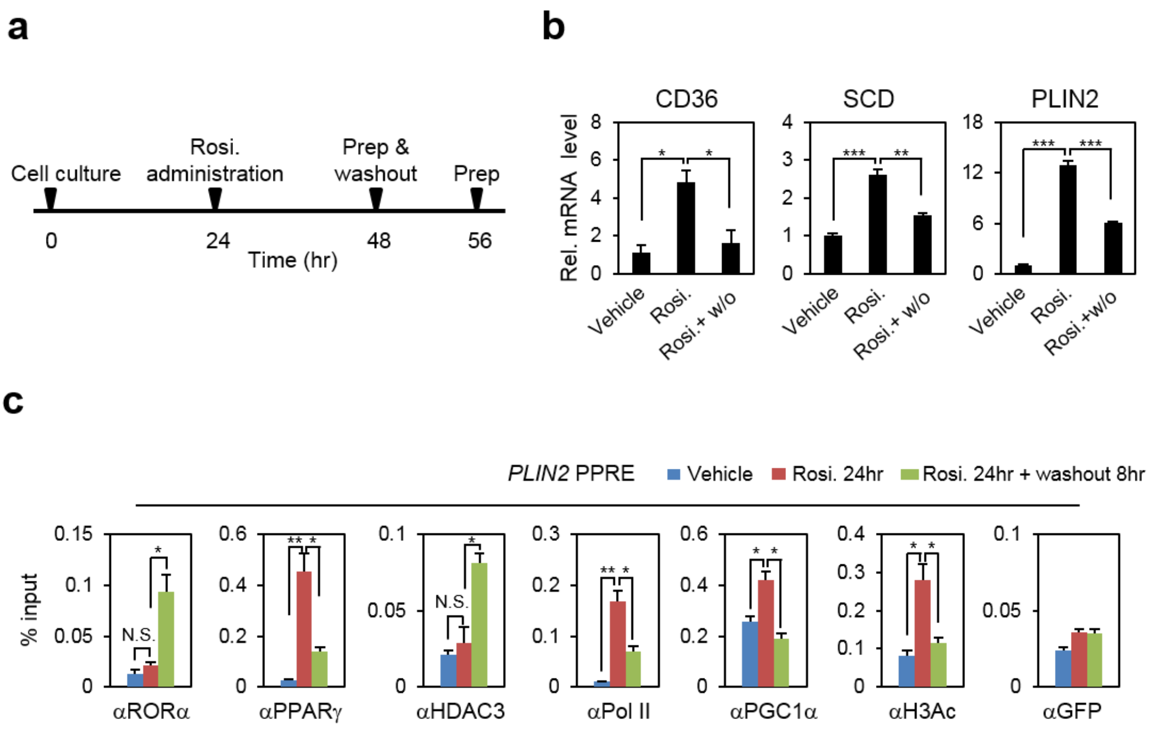

Supplementary Figure 5. Gene expression profile and ChIP assay in response to Rosi.+washout experimental conditions.

(**a**) Schematic representation of experimental design. (**b**) Expression levels of PPARγ target genes in the absence or presence of RORα in Hep3B cells with or without Rosiglitazone (20 μM) treatment for 24 hr and washout 8 hr as determined by qRT-PCR. Expression was normalized to HPRT expression. Data expressed as mean ± SEM. Statistical analysis was performed using one-way ANOVA followed by Tukey's post hoc analysis. *p<0.05, **p<0.01, ***p<0.001. (**c**) ChIP assays were performed on the *PLIN2* promoters in Hep3B cells with or without Rosiglitazone (20 μM) treatment for 24 hr and washout 8 hr. Promoter occupancy of RORα, PPARγ, HDAC3, Pol II, PGC1α, H3Ac and GFP was analyzed. Data expressed as mean ± SEM. Statistical analysis was performed using one-way ANOVA

followed by Tukey's post hoc analysis. *$p<0.05$, **$p<0.01$, ***$p<0.001$, NS=Non-Significant.

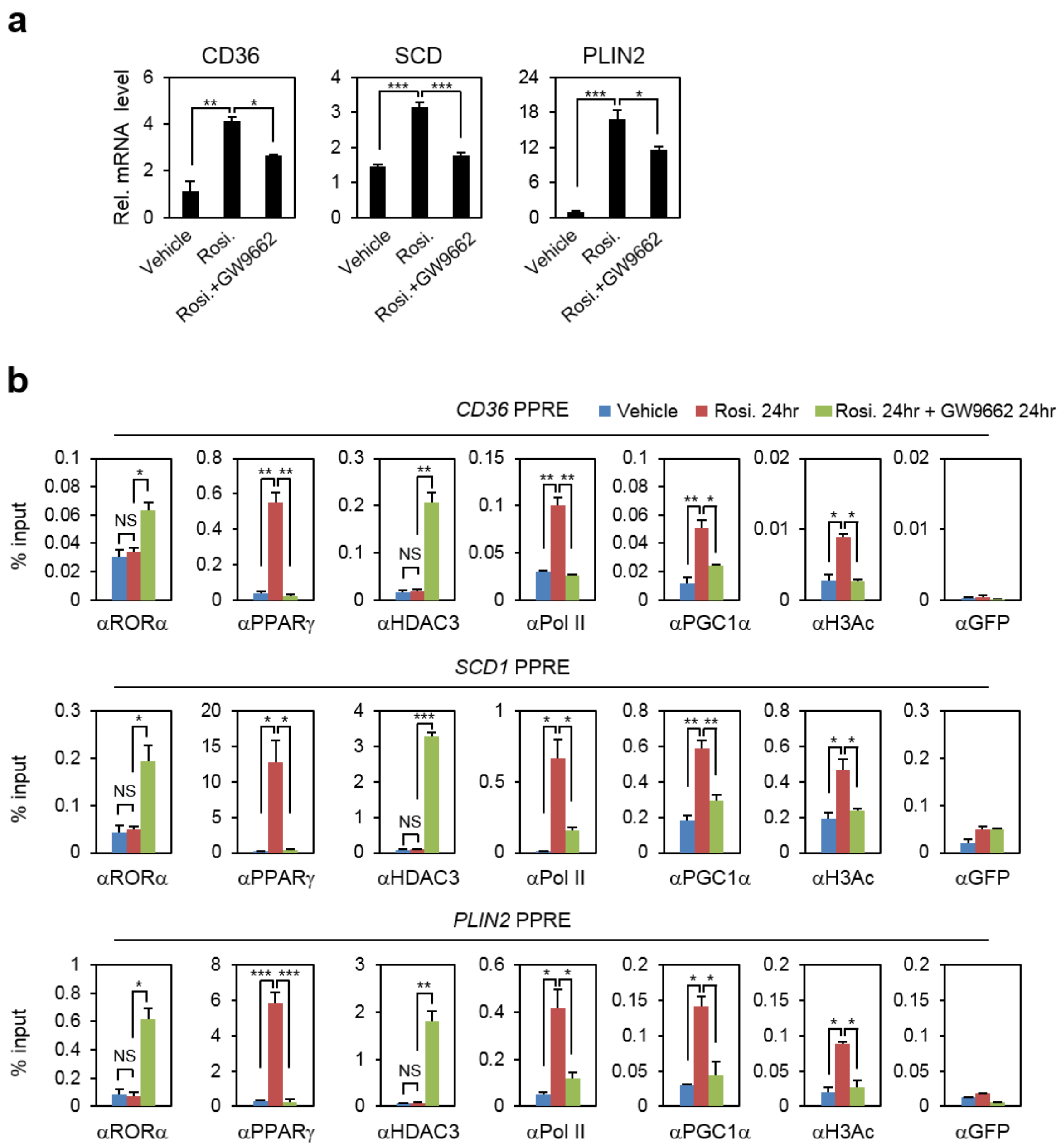

Supplementary Figure 6. The recruitment of RORα to PPARγ target genes promoter increases when PPARγ signal is reduced and it is opposite to PPARγ recruitment.

(**a**) Expression levels of PPARγ target genes in Hep3B cells with or without Rosiglitazone (20 μM) treatment for 24 hr and after treated GW9662 24 hr as determined by qRT-PCR.

Expression was normalized to HPRT expression. Data expressed as mean ± SEM. (**b**) ChIP assays were performed on the *CD36, SCD* and *PLIN2* promoters in Hep3B cells with or without Rosiglitazone (20 μM) treatment for 24 hr and after treated GW9662 24 hr. Promoter occupancy of RORα, PPARγ, HDAC3, Pol II, PGC1α, H3Ac and GFP was analyzed. Data expressed as mean ± SEM. Data expressed as mean ± SEM. Statistical analysis was performed using one-way ANOVA followed by Tukey's post hoc analysis. *p<0.05, **p<0.01, ***p<0.001.

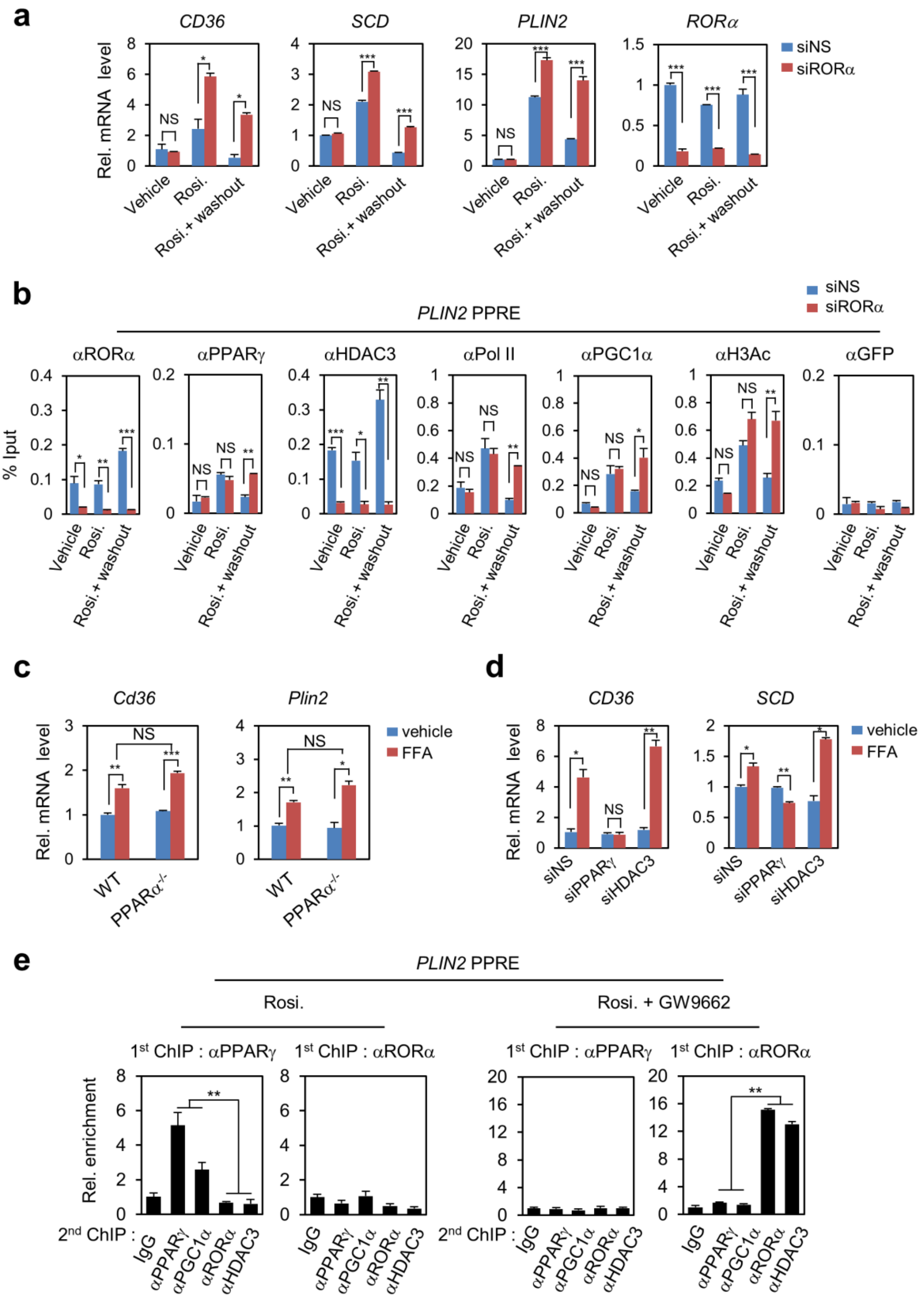
a
CD36
SCD
PLIN2
RORα
Rel. mRNA level
siNS
siRORα
NS
Vehicle
Rosi.
Rosi.+ washout
b
PLIN2 PPRE
siNS
siRORα
αRORα
αPPARγ
αHDAC3
αPol II
αPGC1α
αH3Ac
αGFP
% Iput
Vehicle
Rosi.
Rosi.+ washout
c
Cd36
Plin2
vehicle
FFA
Rel. mRNA level
NS
WT
PPARα-/-
d
CD36
SCD
vehicle
FFA
Rel. mRNA level
siNS
siPPARγ
siHDAC3
e
PLIN2 PPRE
Rosi.
Rosi. + GW9662
1st ChIP : αPPARγ
1st ChIP : αRORα
Rel. enrichment
2nd ChIP :
IgG
αPPARγ
αPGC1α
αRORα
αHDAC3

Supplementary Figure 7. RORα is required to recruit HDAC3 to PPARγ target genes promoter.

(**a**) Expression levels of PPARγ target genes and RORα in the absence or presence of RORα in Hep3B cells with or without Rosiglitazone (20 μM) treatment for 24 hr and washout 8 hr as determined by qRT-PCR. Expression was normalized to HPRT expression. Data expressed as mean ± SEM. Statistical analysis was performed using Student's unpaired t-test. *p<0.05, **p<0.01, ***p<0.001, NS=Non-Significant. (**b**) ChIP assays were performed in the absence or presence of RORα on *PLIN2* promoters in Hep3B cells with or without Rosiglitazone (20 μM) treatment for 24 hr and washout 8 hr. Promoter occupancy of RORα, PPARγ, HDAC3, Pol II, PGC1α, H3Ac and GFP was analyzed. Data expressed as mean ± SEM. Statistical analysis was performed using Student's unpaired t-test. *p<0.05, **p<0.01, ***p<0.001, NS=Non-Significant. (**c**) Expression levels of PPARγ target genes in primary hepatocyte from WT and PPARα$^{-/-}$ mice with or without free fatty acid (FFA: Oleic acid 200 μM and Palmitic acid 100 μM) treatment for 36 hr as determined by qRT-PCR. Expression was normalized to 36B4 expression. Data expressed as mean ± SEM. Statistical analysis was performed using two-way ANOVA. *p<0.05, **p<0.01, ***p<0.001, NS=Non-Significant. (**d**) Expression levels of PPARγ target genes in the absence or presence of PPARγ/HDAC3 in Hep3B cells with or without fatty acid (FFA: Oleic acid 200 μM and Palmitic acid 100 μM) treatment for 24 hr as determined by qRT-PCR. Expression was normalized to HPRT expression. Data expressed as mean ± SEM. Statistical analysis was performed using Student's unpaired t-test. *p<0.05, **p<0.01, NS=Non-Significant. (**e**) Re-ChIP assays were performed in the absence or presence of RORα on *PLIN2* promoters in Hep3B cells with or without Rosiglitazone (20 μM) treatment for 24 hr and after treated GW9662 24 hr to

determine whether RORα and PPARγ are assembled on the same promoter. Data expressed as mean ± SEM. Statistical analysis was performed using one-way ANOVA followed by Tukey's post hoc analysis. *$p<0.05$, **$p<0.01$, ***$p<0.001$.

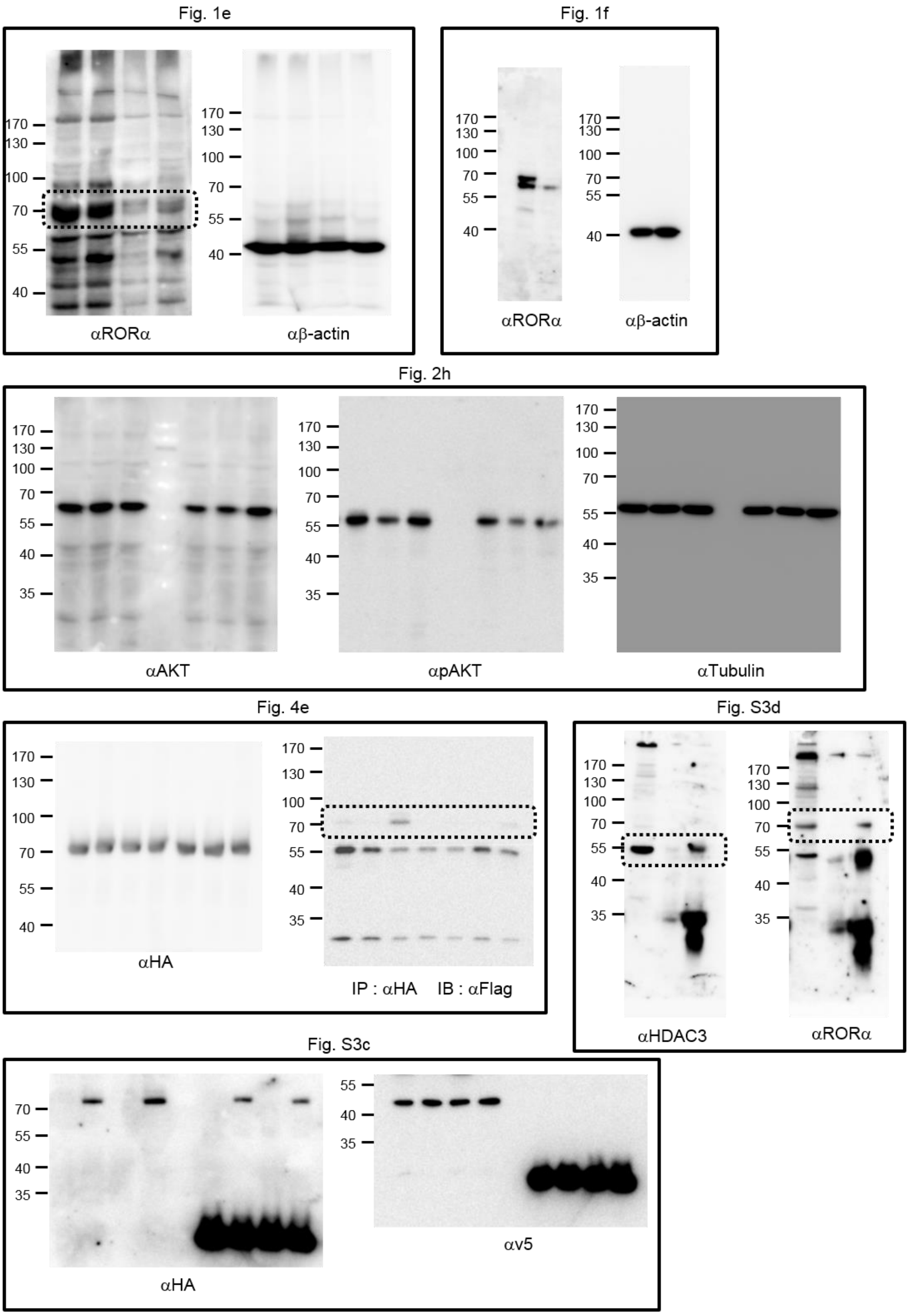

Supplementary Figure 8. Uncropped images for presented immunoblots.
